# From Juvenile Nasopharyngeal Angiofibroma to Nasopharyngeal Carcinoma; A Rare Case Report of Nasopharyngeal Mass

**DOI:** 10.29252/beat-070414

**Published:** 2019-10

**Authors:** Saleh Mohebbi, Mohammad Aghajanpour

**Affiliations:** 1 *Department of Rhinology and Skull Base Surgery, ENT and Head and Neck Research Center, Hazrat Rasoul Hospital, The Five Senses Institute, Iran University of Medical Sciences, Tehran, Iran*

**Keywords:** Juvenile Nasopharyngeal Angiofibroma, Nasopharynx, Carcinoma

## Abstract

Nasopharyngeal masses in young males, first of all presumes Juvenile Nasopharyngeal Angiofibroma (JNA) in the mind, but other benign or malignant tumors should be considered for successful and adequate management of patients and also for minimizing morbidity from unnecessary interventions. We herein present a patient with nasopharyngeal carcinoma (NPC) mimicking JNA. The patient was presumed as JNA and underwent excessive surgical intervention and the final histopathology report was NPC. The aim of our case report is to highlight the importance of complete clinical examination and preoperative imaging in differentiating and ideal management of nasopharyngeal masses.

## Introduction

Nasopharynx can be a source of benign and malignant tumors. The most common nasopharyngeal malignancy is nasopharyngeal carcinoma(NPC), which originates from nasopharynx epithelium. NPC is 0.22 % of all tumors in North America. This tumor may appear as a hearing loss or neck mass [1,2]. The tumor disrupts the performance of the Eustachian tube and it may cause serous otitis media. Due to extensive nasopharyngeal lymphatic drainage that connects the neck, it can cause unilateral neck lymphadenopathy in 44% and bilateral in 22% of cases at the time of diagnosis [3].

Nasal obstruction and epistaxis can also occur. Cranial nerve involvement may also occur due to the spread of the tumor [4,5]. So, the patient with nasopharyngeal carcinoma may exhibit certain signs; however, some cases may be presented with an unusual sign, which can be helpful in dealing with other patients [6]. Therefore, we report this patient with nasopharyngeal angiofibroma symptoms. Ultimately, after the surgery, the diagnosis of nasopharyngeal carcinoma was performed.

## Case Report

**Fig. 1:**
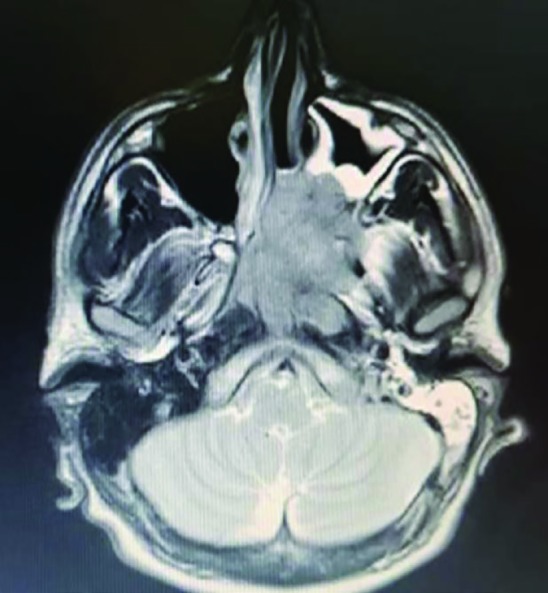
Axial T2-weighted magnetic resonance imaging (MRI) of the patient demonstrating a huge mass in ethmoid, sphenoid and paranasal sinuses invading the maxillary sinus

A 17-year-old boy was referred to the Ear, Nose, Throat outpatient service of Hazrat Rasoul Akam Hospital, Tehran, Iran; with several month history of left side nasal obstruction and intermittent episodes of epistaxis, which gradually increased. Family history was negative. Extra nasal examination showed normal appearance. No cervical lymphadenopathy was detected. Contrast Enhanced Computed Tomography (CT) scan and Magnetic Resonance Imaging (MRI) with gadolinium were obtained which revealed a homogenousely enhancing soft tissue mass lesion widening the pterygopalatine fossae extending into the nasopharynx with bowing the posterior wall of maxillary antrum anteriorly (Figure 1).

**Fig. 2:**
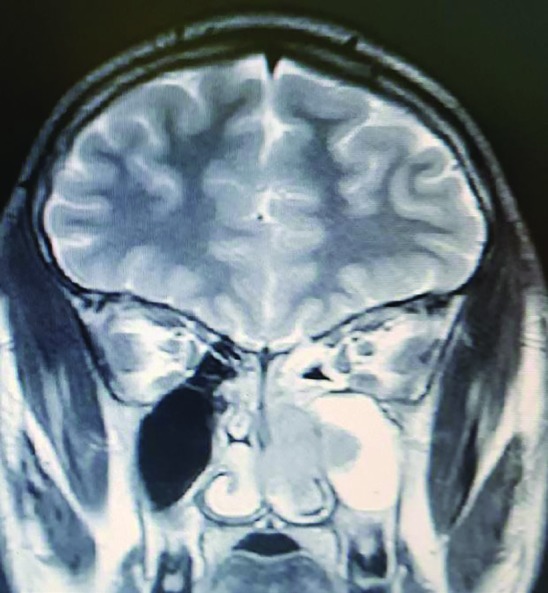
Coronal T2-weighted magnetic resonance imaging (MRI) of paranasal sinuses demonstrating the mass invading the left maxillary sinus

**Fig. 3:**
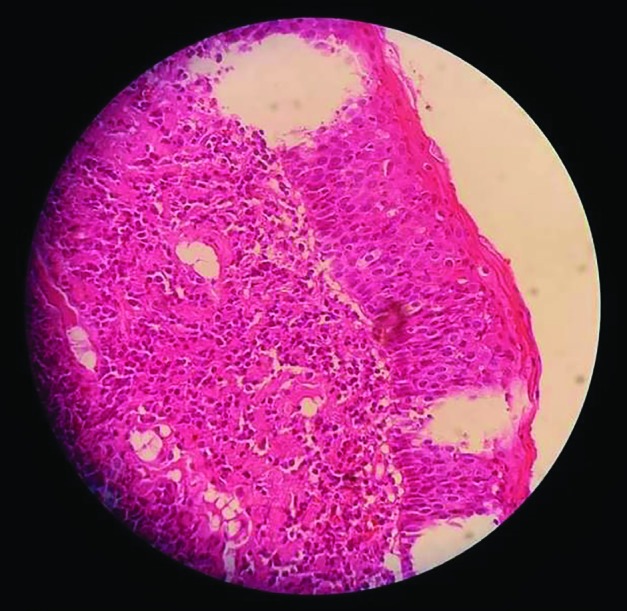
Moderately differentiated squamous cell carcinoma

Invasion into sphenoid sinuses and left infratemporal fossae were also detected (Figure 2). The patient underwent endoscopic sinus surgery. A firm fleshy mass was detected involving left nasal cavity extending into the nasopharynx posteriorly and middle meatus anteriorly; and invasion into the left infratemporal fossae laterally. Some evidence of ethmoid sinus erosion was detected during surgery. Skull base was intact and no intracranial extension was observed. Based on the clinical and radiological features it was initially thought to be Juvenile Nasopharyngeal Angiofibroma (JNA) but when the mass was removed by endoscopic surgery; histopathological examination revealed moderately differentiated squamous cell carcinoma (Figure 3). The patient was subsequently referred for chemoradiation. The patient and his legal guardian provided appropriate informed written consent for publication of the case and the images. 

## Discussion

The diagnosis of JNA is based on clinical and radiological examination and the final proof is histologic. NPC although rare in children but has a predilection for adolescents [1]. Malignant tumors of the nasopharynx are rare and are detected for 1 to 3 percent of all malignancies in children [7]. Nasopharyngeal carcinoma (NPC) accounts for only 20 – 50 % of nasopharyngeal malignancies [4,5]. This is compared with adults, which accounts for almost all nasopharyngeal malignancies. About 5 to 12 % of NPCs occur before the age of 30 [8]. This tumor occurs in boys twice as much as girls. Children between 10 to 19 years old are the most common age group affected by NPC. The genetic and environmental factors of these children are at risk for NPCs [9]. Usually believing NPC in very young people is difficult and that is why the diagnosis is missed or delayed until the patient presents at a locally advanced stage [3].

Non-specificity of symptoms besides neglecting the entity for differential diagnosis might be other reason for a delayed NPC in a young patient. The most common symptom of NPC is a painless cervical mass (70-90%), which is bilateral in 50% of cases [10]. Although on imaging, NPC appears as a heterogeneously enhancing mass arising from nasopharyngeal mucosa with invasion to surrounding structures but also extension into the pterygopalatine fossa and widening it, involvement of infratemporal and masticator spaces may be seen during NPC progression. So NPC may mimick JNA or other benign and malignant tumors in imaging [7]. The possibility of nasopharyngeal malignancies in any child with progressive nasal obstruction, when sign and symptoms persists for more than three weeks despite treatment, and when otitis media occurs unilaterally with cervical lymphadenopathy should be considered. Both CT and MR methods are used to evaluate NPC before surgery. Six percent NPCs have not been identified in endoscopy [11]. In CT scan, 82% of the NPC arises in the posterolateral recess of the pharyngeal wall and 12% appears in the midline [6,7]. In CT and MR imaging, childhood NPCs usually indicate a homogenous enhancement of primary tumor with metastatic lymph nodes [12]. Benign lesions, malignancies and inflammatory conditions are the differential diagnosis of nasopharyngeal masses in children. Of benign tumors, juvenile nasopharyngeal angiofibroma (JNA) is the most important differential diagnosis [13]. In both imaging methods, homogenous enhancement such as JNA can be distinguished from the nasopharyngeal malignancies [14]. JNA which forms less than 1% of the head and neck neoplasms, is an uncommon, benign, fibrovascular tumor. Although the beginning of this damage is not obvious, but according to some researchers, it seems that this damage is usually found in the pterygopalatine cavity and spreads through the sphenopalatine canal to the nasal cavity [11]. In previous studies, only a similar report with our patient was found in 2003 [15]. Therefore, this patient has been reported due to different manifestations, clinical signs and imaging of nasopharyngeal carcinoma.

In conclusion, it is necessary that preoperative imaging of patients suspected for JNA be carefully overviewed to ensure precise diagnosis and accurate management. At the same time, an accurate correlation between clinical and radiological findings is necessary for the best diagnosis.

## Conflict of Interest:

None declared.
